# *Salvia zayuensis* and *Salvia toxantha* (Lamiaceae), two new species from the Hengduan Mountains, China

**DOI:** 10.3897/phytokeys.280.208592

**Published:** 2026-10-19

**Authors:** Yan-Bo Huang, Chu-Hang Lin, Zi-Ning Guo, Xiao-Li Chen, Di-Shen Shi, Yan Luo, Qi Wang, Feng-Luan Liu

**Affiliations:** 1 Key Laboratory of National Forestry and Grassland Administration on East China Plant Conservation and Utilization, Shanghai Chenshan Botanical Garden, Shanghai, 201602, China Key Laboratory of National Forestry and Grassland Administration on East China Plant Conservation and Utilization, Shanghai Chenshan Botanical Garden Shanghai China; 2 State Key Laboratory of Genome and Multi-omics Technologies, BGI Research, Shenzhen, 518083, Guangdong, China State Key Laboratory of Genome and Multi-omics Technologies, BGI Research Shenzhen China; 3 College of Life Sciences, University of Chinese Academy of Sciences, Beijing, 100049, China College of Life Sciences, University of Chinese Academy of Sciences Beijing China

**Keywords:** Chloroplast genome, *
Glutinaria
*, Hengduan Mountains, Lamiaceae, new taxon, sage, *
Salvia
*, taxonomy

## Abstract

Two new species of *Salvia zayuensis* and *S. toxantha* (Lamiaceae), from Southwest China, are described. *Salvia zayuensis* is similar to *S. cyclostegia* but is distinguished by its larger cauline leaves (18.2–24.0 cm), densely hoary appressed trichomes on young rachises and abaxial leaf and bract surfaces, and peach-shaped juvenile inflorescences maturing into 40 cm long. Its corolla is bicolored (purplish-brown upper lip, yellow lower lip) with the anther connective 0.15 cm shorter in arc length. *Salvia toxantha* resembles *S. smithii* in leaf shape, inflorescence structure, bract and bracteole morphology, and corolla color, yet it is characterized by the absence of lemon scent, longer pedicels and calyxes, a slender, bow-shaped corolla, and purple-brown spots on the middle lobe of lower lip. Additionally, its anther connective is ca. 0.4 cm shorter than that in *S. smithii*. Phylogenetic analysis of chloroplast genome places both new species within the subclade Eurysphace of subg. *Glutinaria*.

## Introduction

The genus *Salvia* L. comprises approximately 1,000 species, primarily distributed across East Asia, the Mediterranean, southern Africa, and Central and South America (Drew et al. 2017; Kriebel et al. 2019). Species of *Salvia* have diverse applications, including medicinal, culinary, ornamental, essential oils, and cosmetics (Sharifi-Rad et al. 2018; Masood 2022; Ertas et al. 2023). Since the genus was first established by Bentham (1832–1836), its infrageneric classification and the delimitation of certain species have undergone various revisions; however, the fundamental framework of four or eight subgenera has largely remained (Stibal 1934; Wu et al. 1977). Recent molecular studies have indicated that *Salvia* s.l. is a polyphyletic genus, which can be divided into 11 subgenera (Kriebel et al. 2019). Among these, subg. *Glutinaria* is mainly distributed in East Asia (Hu et al. 2018). This subgenus contains nearly 100 species, mostly found in China, Japan, and the Korean Peninsula (Xiang et al. 2016).

China represents a major center of diversity for East Asian *Salvia*. The “Flora of China” documented 82 species and 24 varieties (Li and Hedge 1994), followed by the subsequent publication of 10 new species (Hu et al. 2014; Huang et al. 2024; Qiu et al. 2024). The Hengduan Mountains represent the center of diversity for *Salvia* in China, hosting 44 species and 12 varieties—accounting for 52.8% of the national total (Wei et al. 2015)—making this region a focal point for botanical surveys of the genus. In April 2019, during a field survey in Zayu County, Xizang Autonomous Region (at the western end of the Hengduan Mountains, China), we encountered a taxon of uncertain identity resembling *S. cyclostegia*. However, it possessed larger leaves, and its young stems, leaf and bract undersides were densely covered with grayish-white tomentose. Notably, the early inflorescence was enclosed by broad-ovate bracts, forming a (long) peachy shape. Subsequently, during an investigation in August 2023 in the northern section of the Hengduan Mountains (Songpan County, Sichuan Province, China), another taxon exhibited some morphological similarities to *S. smithii* was discovered. This taxon lacks the lemonous odor, features a distinctively curved “bow-shaped” corolla, and possesses shorter stamens. Phylogenetic analysis based on chloroplast genome confirmed these two taxa to be new species. Herein, we provide a formal description of these two new species and investigate their phylogenetic positions.

## Materials and methods

### Sample collection and morphological analysis

*Salvia zayuensis* was first discovered in 2019 in Zayu County, Nyingchi, Xizang Autonomous Region, China, with subsequent surveys conducted in 2020 and 2021. *Salvia toxantha* was found in 2023 in Songpan County, Aba Tibetan and Qiang Autonomous Prefecture, Sichuan, China. Following the “Flora of China” (Li and Hedge 1994), key morphological characters were measured and described *in situ*, including habitats, roots, stems, leaves, calyx, corolla, and nutlets. Three and two specimens were prepared for *S. zayuensis* and *S. toxantha*, respectively, and deposited at Shanghai Chenshan Herbarium (**CSH**). Additionally, ten seedlings of each species were collected and cultivated in the National Forest Genetic Resource Bank of Lamiaceae Plants in Shanghai. Morphological comparisons were performed between *S. zayuensis* and its congener *S. cyclostegia*, and between *S. toxantha* and *S. smithii*. The conservation status was preliminarily assessed following the IUCN Red List Categories and Criteria (IUCN 2024).

### Phylogenetic analysis

Phylogenetic trees for both new species were constructed using the maximum likelihood method based on chloroplast genome sequences. The phylogenetic dataset included 18 species 27 accessions primarily belonging to subg. *Glutinaria*; two congeners (*S. cyclostegia* and *S. smithii*) three accessions; 6 distantly related species 11 accessions from subg. *Calosphace*, subg. *Audibertia*, and subg. *Salvia* (Table 1). Whole genome sequencing (WGS) was performed using the Illumina HiSeq platform on seven accessions (four of the two new species and three of their congeners), which were marked with asterisks in Table 1. Chloroplast genomes were assembled by GetOrganelle V.1.7.7.1 (Jin et al. 2020), and uploaded to the National Genomics Data Center (NGDC, https://www.cncb.ac.cn) (Table 1). Furthermore, chloroplast sequences of 38 other accessions, including *S. aerea*, *S. hispanica*, *S. splendens*, were retrieved from the National Center for Biotechnology Information database (NCBI, https://www.ncbi.nlm.nih.gov). Maximum likelihood analysis was conducted using IQ-TREE v2.2.0 (Nguyen et al. 2015) with 1,000 bootstrap replicates. The computation process utilized adaptive multithreaded parallel processing (Threads=AUTO), then the final phylogenetic tree was visualized and enhanced using Interactive Tree Of Life (https://itol.embl.de/itol.cgi) (Letunic and Bork 2021).

**Table 1.** Species information for phylogenetic analyses and NCBI and NGDC numbers.

**No**.	**Species**	**Accession No**.	**No**.	**Species**	**Accession No**.
1	*Salvia aerea*	MW752209.1	23	*Salvia petrophila*	MN520022.1
2	*Salvia aerea*	NC_067739.1	24	*Salvia petrophila*	NC_050899.1
3	*Salvia bulleyana*	MH603954.1	25	*Salvia prionitis*	MZ666404.1
4	*Salvia bulleyana*	NC_041092.1	26	*Salvia prionitis*	NC_065866.1
5	*Salvia campanulata*	MT742542.1	27	*Salvia przewalskii*	MH603953.1
6	*Salvia campanulata*	NC_057284.1	28	*Salvia przewalskii*	NC_041091.1
7	*Salvia castanea*	MW387501.1	29	*Salvia roborowskii*	MK944406.1
8	*Salvia chienii*	OK094518.1	30	*Salvia roborowskii*	MW752206.1
9	*Salvia chinensis*	PV591959.1	31	*Salvia sikkimensis*	NC_071851.1
10	*Salvia cyclostegia*	C_AA489590*	32	*Salvia sikkimensis*	OP186468.1
11	*Salvia deserta*	MT156378.1	33	*Salvia smithii*	C_AA489591*
12	*Salvia deserta*	MZ461466.1	34	*Salvia smithii*	C_AA489592*
13	*Salvia digitaloides*	PV235598.1	35	*Salvia sonchifolia*	MW752201.1
14	*Salvia grandifolia*	MZ461467.1	36	*Salvia sonomensis*	PV870868.1
15	*Salvia grandifolia*	NC_084046.1	37	*Salvia splendens*	MN520024.1
16	*Salvia hispanica*	MN520017.1	38	*Salvia splendens*	OM617847.1
17	*Salvia hispanica*	MT083896.1	39	*Salvia toxantha* sp. nov.	C_AA489593*
18	*Salvia japonica*	MW381778.1	40	*Salvia toxantha* sp. nov.	C_AA489594*
19	*Salvia miltiorrhiza*	MT012420.1	41	*Salvia tricuspis*	MW752207.1
20	*Salvia miltiorrhiza*	OR652279.1	42	*Salvia tricuspis*	NC_067738.1
21	*Salvia officinalis*	MZ636550.1	43	*Salvia zayuensis* sp. nov.	C_AA489595*
22	*Salvia officinalis*	PV340580.1	44	*Salvia zayuensis* sp. nov.	C_AA489596*

## Results

### Taxonomic treatment

#### Salvia zayuensis

Taxon classificationPlantaeLamialesLamiaceae

Yan.B.Huang & Feng.L.Liu sp. nov.

078323D4-D87D-5B4B-AD65-32D6D9558537

urn:lsid:ipni.org:names:77382198-1

Fig. 1

##### Type.

China • Xizang Autonomous Region: Nyingchi City, Zayu County, Shangchayu Town, 28°52'41"N, 96°04'06"E, 2032 m, 16 April 2021, *Huang et al., S1722* (Holotype: CSH, CSH0188553!; isotypes: CSH).

**Figure 1.**
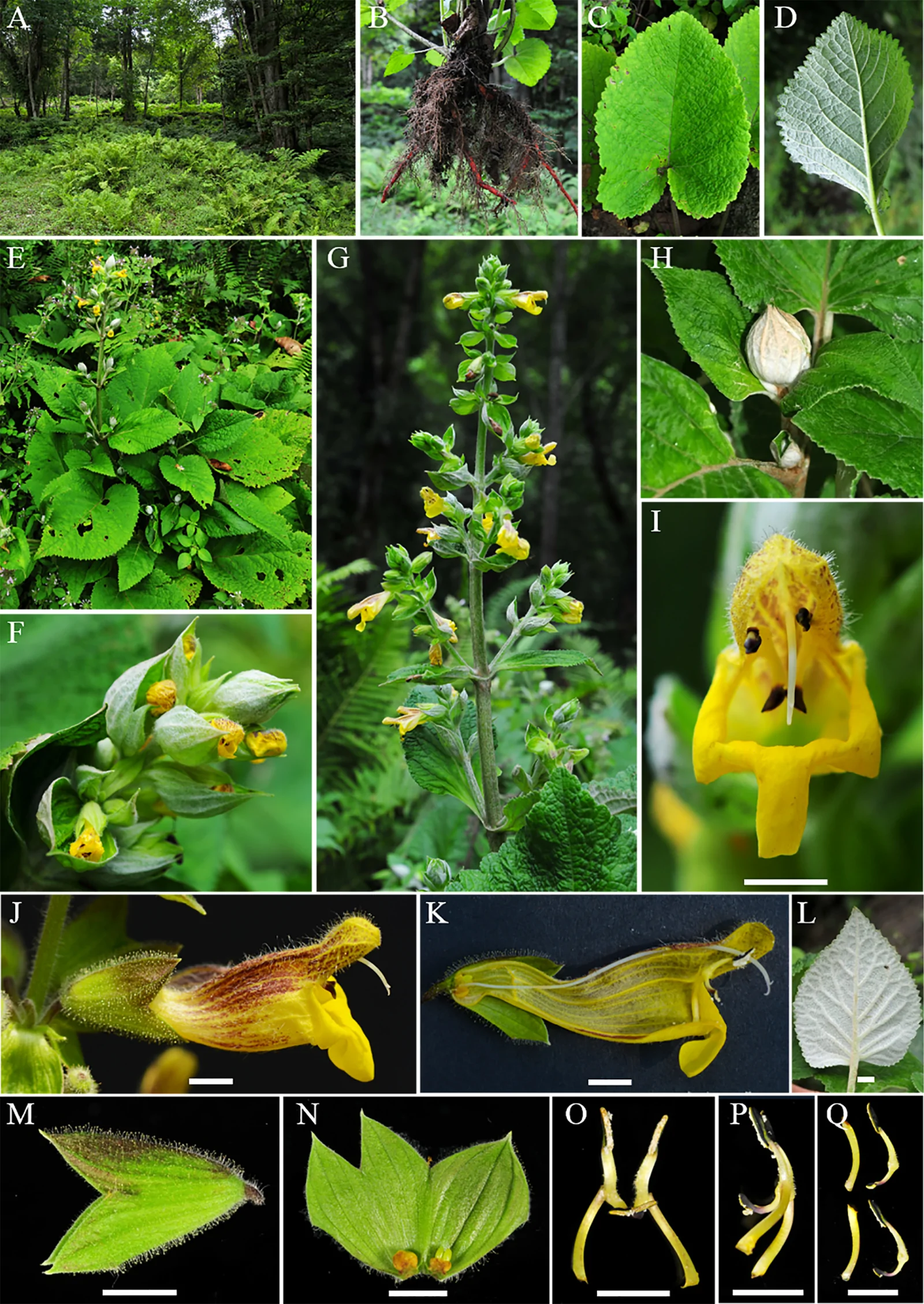
*Salvia zayuensis* sp. nov. **A**. Habitat; **B**. Fleshy root; **C**. Leaf (adaxial surface); **D**. Leaf (abaxial surface); **E**. Flowering plant; **F**. Terminal inﬂorescence with ovate bracts; **G**. Inﬂorescence; **H**. Peach-shaped initial inflorescence enclosed by bracts; **I**. Flower (front view); **J**. Flower (side view); **K**. Flower (longitudinal section); **L**. Young leaves with pubescent abaxial surface; **M**. Calyx (side view); **N**. Calyx (spreading inner surface); **O**. Stamens (front view); **P**. Stamens (side view); **Q**. Disassembled stamens. Scale bar: 5 mm.

##### Diagnosis.

*Salvia zayuensis* exhibits morphological similarities to *S. cyclostegia* in the arrangements and shapes of leaf and bract, calyx and corolla characteristics, and filament length. However, *S. zayuensis* can be distinguished by larger leaves, with stem leaves 18.2–24.0 cm long; densely hoary appressed trichomes (felted puberulent) on young rachises and abaxial leaf and bract surfaces; peach-shaped inflorescences when young, enveloped by several layers of broadly ovate bracts; longer inflorescences (40 cm), with several branches; bicolored corollas, with the upper side purplish-brown, lower side predominantly yellow; anther connectives with an arc length 0.15 cm shorter; later flowering period, from August to September (vs. March to April) (Table 2).

**Table 2.** Main morphological variations of *S. zayuensis* sp. nov. and its morphologically similar taxon.

**Organs**	**Characters**	***S. zayuensis***	***S. cyclostegia***
**Plant**	Height (cm)	≤100	≤40
**Basal leaf**	Petiole length (cm)	9.1–13.1	2.3–8.0
	Blade length × width (cm)	10.0–18.2 × (8.7–18.3)	(6.8–12.5) × (3.7–6.7)
**Cauline leaf**	Petiole length (cm)	4.5–13.5	Shorter to nearly absent
	Blade length × width (cm)	(18.2–24.0) × (2.6–18.0)	(2.8–7.5) × (2.2–4.0)
**Basal / cauline leaf**	Indumentum	Adaxial surface sparsely tomentellous; abaxial surface densely hoary-pubescent, particularly along the veins	Both surfaces densely glandular-punctate, veins sparsely pilose
**Inflorescence**	Type	Racemiform panicle; young inflorescence (long) peach-shaped	Racemes predominant
	Branch number	Usually	Occasionally
	Length (cm)	≤40	≤20
	Shape of the lowermost bract	Foliaceous, long ovate	Subcircular
	Size of the lowermost bract (cm)	(11.7–17.0) × (4.5–6.0)	1.0–2.0 in diameter
	Indumentum	Young rachis and abaxial bract surface densely hoary pannose	Young rachis and abaxial bract surface villous
**Flower**	Corolla color	Bicolorous, dorsally purplish brown, middle and ventrally mostly yellow	Yellowish-white, pale yellow
	Arc length of anther connective (cm)	0.60–0.67; mean value = 0.64	0.68–0.89; mean value = 0.79
	Flowering period	August to September	April to May

##### Description.

***Plant*** perennial herb to 100 cm tall. ***Roots*** taproot thick, succulent, cylindrical, 6.3–23.2 cm long; fibrous roots numerous. ***Stems*** quadrangular, angled upward, branched from base. ***Leaves*** both basal and cauline, blade oval, apex blunt, base cordate, margin obtuse crenate; adaxial surface green, sparsely tomentellous, abaxial surface densely hoary pannose, especially along the veins. Basal leaves: petiole 9.1–13.1 cm; blade 10.0–18.2 cm long, 8.7–18.3 cm wide. Stem leaves: petiole 4.5–13.5 cm; blade 18.2–24.0 cm long, 12.6–18.0 cm wide. ***Inﬂorescence*** racemiform panicle, up to 40 cm in length, branched, rachis puberulent; young inﬂorescences enclosed by tomentose bracts, forming (long) peach shape; 2–6 flowered verticillasters. ***Bracts*** adaxial surface green; abaxial surface densely hoary tomentose. First pair of basal rachis leaf–like, elongate–ovate, apex acuminate, length 11.7–17.0 cm, width 4.5–6.0 cm. Terminal bracts broadly ovate, length 3.0–4.4 cm, width 2.0–2.5 cm. ***Bracteoles*** broadly lanceolate, ca. 0.5 × 0.2 cm. ***Pedicels*** 0.40–0.50 cm, densely villous. ***Calyx*** broadly campanulate, length 1.4–1.6 cm, externally densely glandular hairs. Upper lip brownish anteriorly, lower lip green; upper lip entire with 3 veins; lower lip slightly longer, 2–toothed, teeth triangular, aristulate. ***Corolla*** bicolored, upper side purple–brown, lower side predominantly yellow, 2.8–3.4 cm long. Corolla tube 2.2–2.6 cm long, sparsely pubescent. Cylindrical at base, ventrally dilated in middle, overall ‘Z’–shaped. Pilose annulate 0.5–0.7 cm from the base internally. Upper lip galeate, 0.7–0.8 cm long, purple–brown spots and densely villous externally. Lower lip 0.5–0.6 cm long, 3 shallow lobes: middle lobe broad, 0.6 cm long and 0.8–0.9 cm wide; side lobes small and semicircular. ***Stamens*** curved, linear length 0.60–0.73 cm, locally glandular hairs. Arc lengths of anther connectives 0.60–0.67 cm. Upper arms ca. 0.37 cm, lower arms ca. 0.26 cm; both anthers fertile, upper arms hidden within the upper lip, lower anthers smaller, fused. ***Style*** 2.9–3.2 cm long, 0.2–0.3 cm exserted; stigma unequal 2–lobed, posterior shorter. ***Filaments*** cylindrical, ca. 0.7 cm long, glabrous. ***Nectariferous disc*** milky yellow, slightly swollen. ***Nutlets***: long ovate, light yellowish–brown, 0.32–0.38 × 0.20–0.26 cm.

##### Phenology.

Flowering from August to September, and fruiting one month later.

##### Etymology.

The specific epithet is derived from the type locality, Zayu County, Nyingchi City, Xizang Autonomous Region, China.

##### Vernacular name.

The Chinese name is: zá yú shǔ wěi cǎo (察隅鼠尾草).

##### Distribution and habitat.

*Salvia zayuensis* was recorded in both Shangchayu and Zhuwagen Towns in Zayu County, Xizang Autonomous Region, China, at altitudes ranging from 1920 to 2640 m. It prefers filtered light and thrives in semi-open woodlands, forest edges, and roadsides, where the ground is typically covered with scree and leaf litter. The vegetation consists of coniferous and broad-leaved mixed forests in subtropical montane regions or river valleys.

##### Conservation status.

Eight populations of *S. zayuensis* were recorded in Zayu County, Xizang Autonomous Region, China, totaling approximately 6,000 individuals. Given the robust growth of the species and its unique habitat, it is presumed that additional populations may exist within the region, and it is endemic. Considering the high humidity and minimal anthropogenic disturbance in the area, the new species is tentatively assessed as Least Concern (LC) according to the IUCN Red List Categories and Criteria (2024).

##### Additional specimens examined

**(*Paratypes*)**. China • Xizang Autonomous Region: Nyingchi City, Zayu County, Shangchayu Town, 29°09'59"N, 96°24'53"E, 2450 m, 8 April 2019, *Wang et al., S1207* (CSH).

#### Salvia toxantha

Taxon classificationPlantaeLamialesLamiaceae

Yan.B.Huang & Feng.L.Liu sp. nov.

1EAF832F-C48B-56C6-8D58-ADAC7101BD24

urn:lsid:ipni.org:names:77382199-1

Fig. 2

##### Type.

China • Sichuan Province: Aba Tibetan and Qiang Autonomous Prefecture, Songpan County, Daxing Town, 32°31'18"N, 103°52'5"E, 2,700–2,850 m, 24 August 2023, *Huang et al., S1912* (Holotype: CSH, CSH0206978!; isotype: CSH).

**Figure 2.**
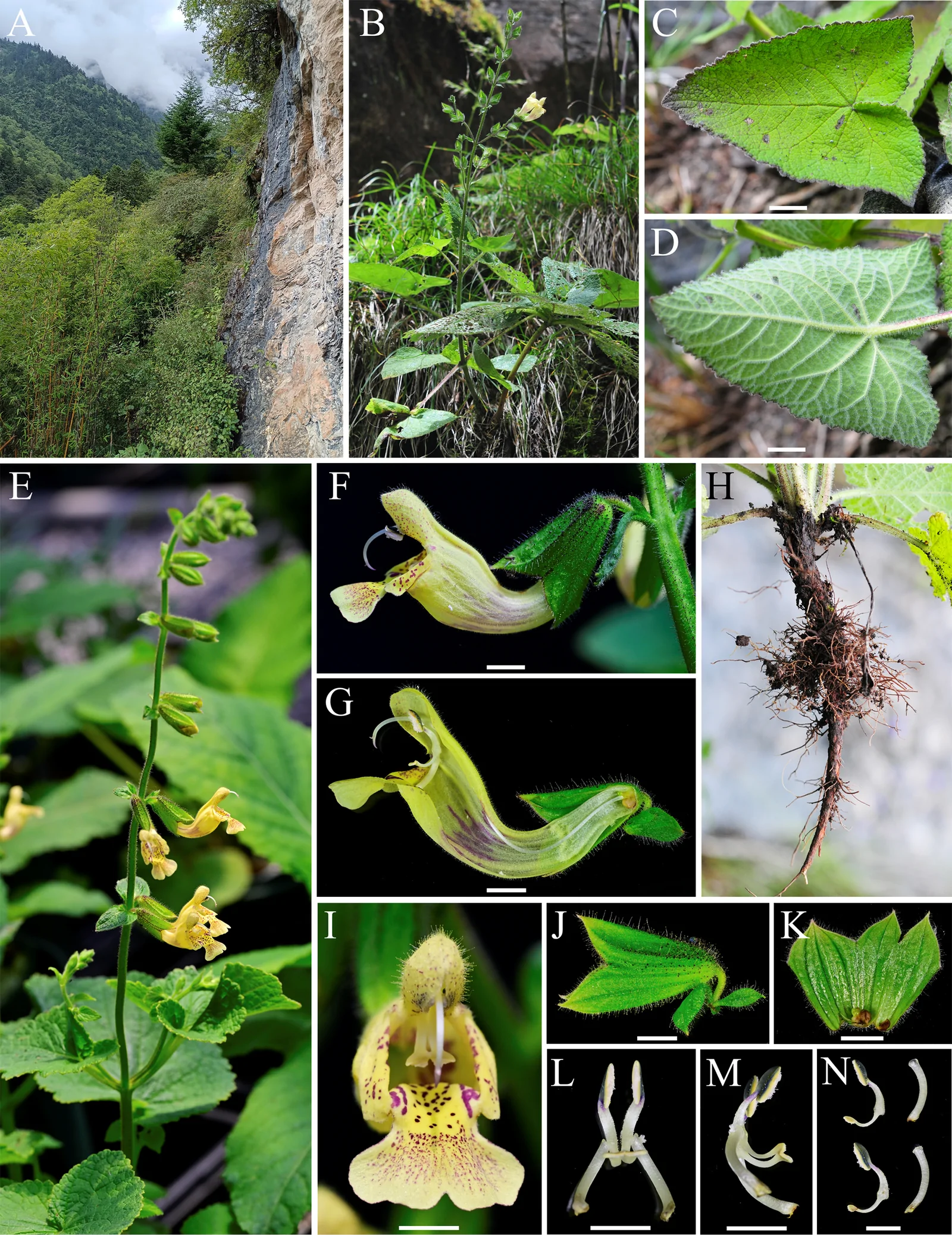
*Salvia toxantha* sp. nov. **A**. Habitat; **B**. Flowering plant; **C**. Leaf (adaxial surface); **D**. Leaf (abaxial surface); **E**. Inﬂorescence; **F**. Flower (side view); **G**. Flower (longitudinal section); **H**. Fleshy taproot; **I**. Flower (front view); **J**. Calyx (side view); **K**. Calyx (spreading inner surface); **L**. Stamens (front view); **M**. Stamens (side view); **N**. Disassembled stamens. Scale bar: 5 mm.

##### Diagnosis.

*Salvia toxantha* is similar to *S. smithii*, displaying high morphological resemblance in leaf shape, inflorescence arrangement, bract and bracteole morphology, flower number of verticillaster, corolla color, purplish-red stripes on the inner middle of the corolla tube, and flowering period. However, *S. toxantha* is distinguished by its lack of lemon scent throughout the entire plant; pedicels and calyxes 0.2–0.3 cm longer; a corolla more slender, distinctly curved in a “bow” shape; and the middle lobe of the lower lip densely covered with purple-brown spots. Additionally, the arc length of anther connective in *S. toxantha* is shorter by ca. 0.4 cm (Table 3).

**Table 3.** Key morphological differences between *S. toxantha* sp. nov. and *S. smithii*.

**Organs**	**Characters**	***S. toxantha***	***S. smithii***
**Plant**	Height (cm)	≤60	≤99
**Leaf**	Odor	Odorless	Lemon-scented
**Flower**	Pedicel length (cm)	0.41–0.62	0.25–0.46
	Calyx length (cm)	1.7–2.0	1.4–1.7
	Corolla length (cm)	4.1–4.8, mean 4.4	3.5–4.3, mean 4.0
	Corolla shape	Gradually dilated from the mid-distally, curved upward like a bow	Ventrally expanded from the middle, slightly curved upward
	Size of corolla tube (cm)	Length 2.8–3.4, mean 3.0; corolla throat 0.58–0.83 cm wide, mean 0.75	Length 2.1–2.4, mean 2.2; corolla throat 0.91–1.1 wide, mean 1.0
	Indumentum of corolla tube	Externally glandular-pubescent	Externally subglabrous
	Arc length of anther connective (cm)	0.68–0.82, mean 0.78	1.02–1.25, mean 1.17

##### Description.

***Plant*** perennial herb, ≤ 60 cm tall. ***Roots*** single or multiple taproots, succulent, 5.5–16.0 cm long; brown fibrous roots dense. ***Stems*** erect, densely pubescent with villose and glandular pilose. ***Leaves*** basal and cauline present. Both petioles often reddish-purple, indumentum as on stem; blades broadly ovate to cordate, apex acute to shortly acuminate, base cordate or hastate, margins finely and densely crenate. Adaxially green, slightly wrinkled, densely appressed-pubescent; margins and veins mixed villose. Abaxially pale green, veins prominent and densely white-villose, with the indumentum denser than on the adaxial surface. Basal leaves with petioles without sheath, 10.0–14.9 cm long, blades 9.5–18.5 cm long, 8.0–11.5 cm wide; cauline leaves with petioles having sparse glandular pilose, 11.8–13.2 cm long; blades 7.0–16.3 cm long, 5.0–15.5 cm wide. ***Verticillasters*** 2-flowered, loosely arranged, racemes or panicles. ***Bracts*** pale green, ovate to long-ovate, apex acute, base cuneate, entire. Adaxial side with sparse fine hairs; abaxial side glandular, hairs particularly dense along margins. ***Bracteoles*** ovate, apex acute to acuminate; ca. 9 mm long, 6 mm wide. Indumentum similar to bracts. ***Pedicel*** 0.41–0.62 cm, densely villose and glandular pilose. ***Calyx*** campanulate, 1.7–2.0 cm long. Upper lip 3 shallow lobes, lower lip 2 lobes deeply cleft. Teeth triangular, apex acute, purple-red, margins densely glandular pilose. Externally green, densely glandular pilose, gland bases notably darkened on mid-posterior part of the upper lip; internally green, glabrous. ***Corolla*** yellow, 4.1–4.8 cm long, overall distinctly curved upward in a “bow” shape. Tube 2.8–3.4 cm long, throat ca. 0.75 cm wide; base narrowly cylindrical, becoming radially inflated from the mid-distally. External surface with short glandular hairs, internal with purple-brown longitudinal stripes and a hairy annulus 1.2 cm from the base. Upper lip straight, galeate, 1.1–1.2 cm long, external densely glandular-pubescent with sparse purple spots. Lower lip 0.7–0.9 cm long, 1.3 cm wide, 3-lobed; middle lobe large, length × width ca. 0.9 × 1.3 cm, obovate to nearly circular, densely covered with dark purple-brown spots, rarely unspotted; side lobes oblique-ovate, ca. 0.4 cm wide. ***Stamens*** semi-circular, linear length 0.7–0.9 cm. Arc length of anther connective 0.68–0.82 cm, upper and lower arms nearly equal, each ca. 0.38 cm. Upper anthers hidden in the upper lip, lower anthers fused, fertile. ***Style*** 3.8 cm long, exserted. Stigma with 2 unequal shallow lobes, the posterior lobe very short. ***Filaments*** cylindrical, 0.8–0.9 cm long. ***Nectariferous disc*** pale yellow, slightly swollen anteriorly. ***Nutlets*** ovate, gray-brown, 0.35–0.39 cm long, 0.25–0.28 cm wide.

##### Phenology.

Flowering from August to September, and fruiting one month later.

##### Etymology.

Species epithet “toxantha” refers to the arched corolla of this species.

##### Vernacular name.

The Chinese name is: gōng huā shǔ wěi cǎo (弓花鼠尾草).

##### Distribution and habitat.

*Salvia toxantha* is currently known from only a single population, restricted to Hongyanwo, Daxing Town, Songpan County, Sichuan, China. It inhabits shaded limestone cliffs at forest borders or exposed moist cliffs in valleys, at an altitude of ca. 2,800 m. The local vegetation consists of conifer-broadleaf mixed forest in warm temperate subalpine zones.

**Conservation status**. *Salvia toxantha* is limited to one observed distribution site in Hongyanwo (Daxing Town, Songpan, Sichuan, China), with an estimated number of mature individuals between 300 and 500. As field surveys are not yet exhaustive in the entire range, the new species is tentatively classified as Data Deficient (DD) according to the IUCN Red List Categories and Criteria (2024).

##### Additional specimens examined

**(*Paratypes*)**. China • Sichuan Province: Garzê Tibetan Autonomous Prefecture, Daofu County, Shachong Town, 30°47'25"N, 101°32'07"E, 2850 m, 15 August 2011, *Liu et al., 3217* (KUN) · Danba County, Bian’er Town, 31°02'52"N, 101°36'58"E, *Liu et al., FW11240* (KUN).

### Phylogenetic relationships

**Figure 3.**
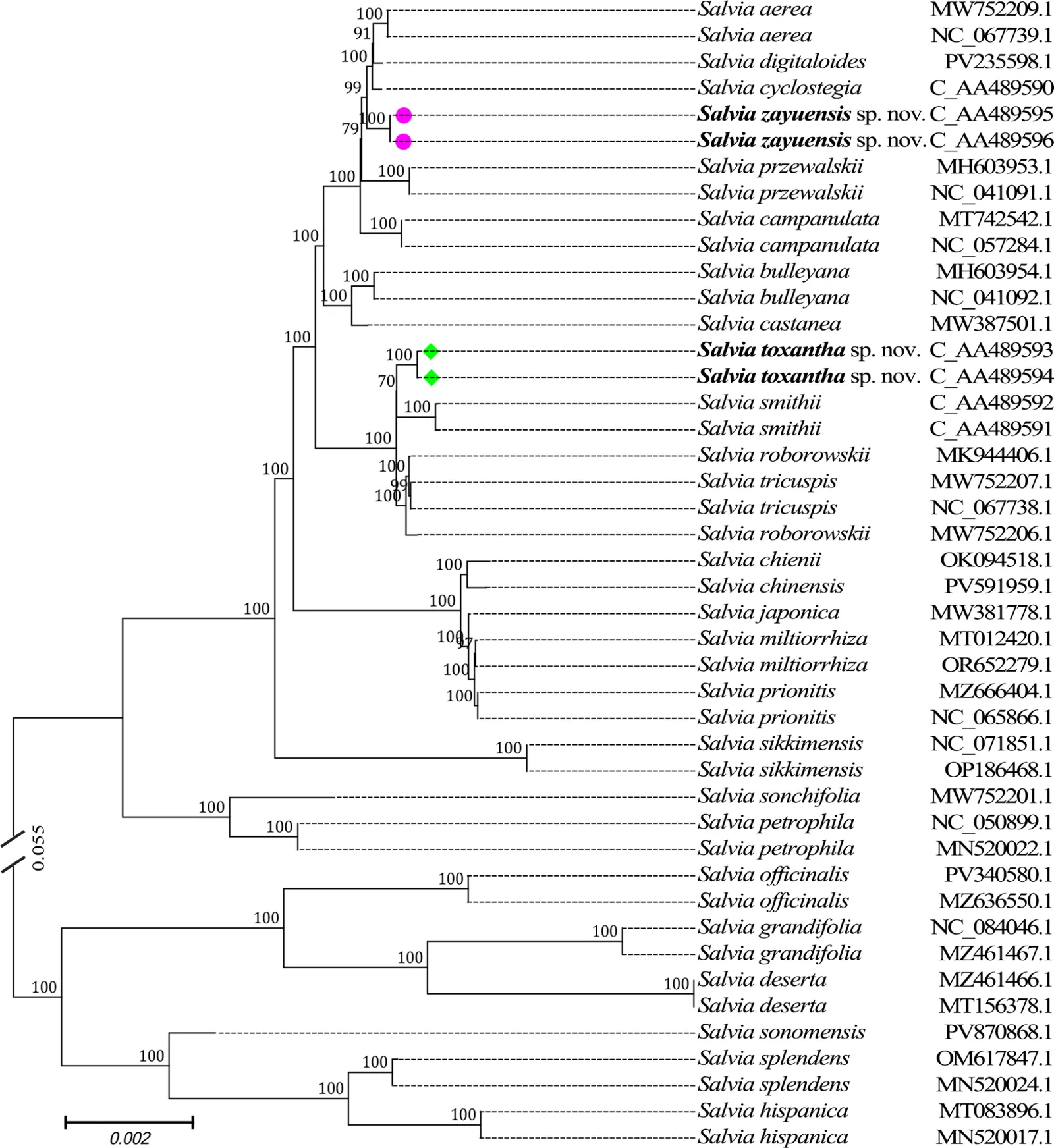
Maximum likelihood tree based on chloroplast genome for *S. zayuensis* and *S. toxantha*.

In the maximum likelihood phylogenetic tree constructed from 44 accessions representing 26 species, the support values for all repeated samples exceeded 97% (Fig. 3), indicating a high reliability of this phylogeny. The phylogenetic topology demonstrated that *S. zayuensis* and *S. toxantha* each independently formed monophyletic clades with strong support (BS = 100%). *Salvia zayuensis* was most closely related to *S. cyclostegia* (BS = 99%), followed by *S. digitaloides* and *S. aerea*. Meanwhile, *S. toxantha* and *S. smithii* are sister to each other (BS = 70%), and this clade is the robustly supported sister to *S. roborowskii* and *S. tricuspis* (BS = 100%).

## Discussion

*Salvia zayuensis* was discovered in the western part of the Hengduan Mountains. It exhibits morphological characteristics such as single leaves, bell-shaped calyx, fused and fertile lower anthers, and a corolla tube swollen ventrally. According to the traditional morphological classification of genus *Salvia* in the “Flora of China” (Li and Hedge 1994), it belongs to sect. *Eurysphace* of subg. *Salvia* sensu Wu et al., (1977). Among the species of sect. *Eurysphace* in the southeastern part of Xizang Autonomous Region, *S. cyclostegia* exhibits the highest affinity to *S. zayuensis*. Other species, such as *S. castanea*, *S. przewalskii*, and *S. campanulata*, differ significantly from *S. zayuensis* in leaf size, bract morphology, corolla color, and pubescence type. Thus, *S. cyclostegia* was considered as the sister taxon of the new species, and this assignment is corroborated by the chloroplast genome phylogeny, which recovered the two species as closely related. In light of its phylogenetic relationship with *S. cyclostegia*, *S. digitaloides*, and *S. aerea* in this study, the new species *S. zayuensis* is nested within the subclade Eurysphace of subg. *Glutinaria* (Hu et al., 2018). This subclade encompasses approximately 75% of the species formerly assigned to subg. *Salvia* sensu Wu et al., (1977). Furthermore, *S. zayuensis* contributes a distinctive inflorescence trait: the primary one is enclosed by ovate, puberulent bracts, forming a peach-like structure. This feature is uncommon in the subclade Eurysphace, in which most species possess small, loosely appressed, or caducous bracts. Consequently, this trait serves as a key diagnostic character for the new species *S. zayuensis*.

*Salvia toxantha* is located in the northern section of the Hengduan Mountains. According to the traditional classification adopted in the “Flora of China” (Li and Hedge 1994), it belongs to sect. Eurysphace of subg. *Salvia* sensu Wu et al. (1977), as does *S. zayuensis*. Morphologically, however, *S. toxantha* most closely resembles *S. smithii* among the species of this section. Our phylogenetic analysis confirmed that *S. toxantha* is sister to *S. smithii*, nested within the subclade Eurysphace of subg. *Glutinaria* (Hu et al., 2018). Nevertheless, the bootstrap support for the branch uniting *S. toxantha* and *S. smithii* is low (BS = 73%), and their sister group falls within another branch of subg. *Glutinaria*, subclade Annua. This may be attributed to incomplete lineage sorting or the absence of currently unknown closely related species of *S. toxantha*, necessitating further validation through expanded taxon sampling and more robust molecular datasets.

The Hengduan Mountains are recognized as the primary center for *Salvia* diversity across China and East Asia (Wei et al. 2015), serving as a critical hotspot for speciation and endemism within the subclade Eurysphace of subg. *Glutinaria* (Wang et al., 2015). The discovery of two new species, *S. zayuensis* and *S. toxantha*, from Zayu and Songpan Counties, respectively, further reinforces these assertions, highlighting the necessity for continued and comprehensive botanical surveys in this region to fully uncover its hidden floral diversity.

## Supplementary Material

XML Treatment for Salvia zayuensis

XML Treatment for Salvia toxantha

## Additional information

### Artificial Intelligence (AI) use

The authors accept full responsibility for the content of the manuscript, including the disclosure of any use of AI.

No AI tools were used in the preparation of this manuscript.

### Author ORCIDs

Y.-B. Huang https://orcid.org/0000-0003-0620-3071

C.-H. Lin https://orcid.org/0009-0000-8393-2732

X.-L. Chen https://orcid.org/0000-0002-4878-1905

D.-S. Shi https://orcid.org/0009-0003-4357-360X

F.-L. Liu https://orcid.org/0000-0002-1027-3278

### Data availability

All of the data that support the findings of this study are available in the main text.
